# A Relação entre Compartimentos de Volume Extracelular e Matriz Metaloproteinase 2 na Remodelação do Ventrículo Esquerdo após o Infarto do Miocárdio

**DOI:** 10.36660/abc.20220061

**Published:** 2022-11-23

**Authors:** Ferhat Eyyupkoca, Nilnur Eyerci, Mehmet Sait Altintas, Mehmet Ali Felekoglu, Halil Ibrahim Biter, Siho Hidayet, Serkan Sivri, Bekir Demirtas, Omer Faruk Ates

**Affiliations:** 1 Dr. Nafiz Korez Sincan State Hospital Departamento de Cardiologia Ankara Turquia Departamento de Cardiologia, Dr. Nafiz Korez Sincan State Hospital, Ankara – Turquia; 2 Departamento de Biologia Médica Kafkas University Faculty of Medicine Kars Turquia Departamento de Biologia Médica, Kafkas University Faculty of Medicine, Kars – Turquia; 3 Istanbul Yedikule Chest Diseases and Thoracic Surgery Training and Research Hospital Departamento de Cardiologia Istanbul Turquia Departamento de Cardiologia, Istanbul Yedikule Chest Diseases and Thoracic Surgery Training and Research Hospital, Istanbul – Turquia; 4 Atakent Hospital Departamento de Cardiologia Yalova Turquia Departamento de Cardiologia, Atakent Hospital, Yalova – Turquia; 5 Istanbul Haseki Training And Research Hospital Departamento de Cardiologia Istanbul Turquia Departamento de Cardiologia, Istanbul Haseki Training And Research Hospital, Istanbul – Turquia; 6 Departamento de Cardiologia Inonu University Faculty of Medicine Malatya Turquia Departamento de Cardiologia, Inonu University Faculty of Medicine, Malatya – Turquia; 7 Kirsehir State Hospital Departamento de Cardiologia Kirşehir Turquia Departamento de Cardiologia, Kirsehir State Hospital, Kirşehir – Turquia; 8 Cankiri State Hospital Departamento de Cardiologia Cankiri Turquia Departamento de Cardiologia, Cankiri State Hospital, Cankiri – Turquia; 9 Sakarya University Faculty of Medicine Departamento de Cardiologia Sakarya Turquia Departamento de Cardiologia, Sakarya University Faculty of Medicine, Sakarya – Turquia

**Keywords:** Infarto do Miocárdio/metabolismo, Remodelação Ventricular Esquerda, Miofibroblastos/citologia, Metaloproteinase da Matriz, Mapeamento TI

## Abstract

**Fundamento:**

As matrizes metaloproteinases (MMPs) podem afetar o volume extracelular (VEC) e seus compartimentos, e isso pode oferecer informações mais detalhadas sobre o mecanismo de remodelação adversa (RA) do ventrículo esquerdo (VE) após o infarto agudo do miocárdio (IM).

**Objetivos:**

Investigar o papel que as alterações (Δ) nos compartimentos de VEC (volume matriz (MVi) e volume celular (CVi)) desempenham no desenvolvimento de RA após o IM, e sua relação com as expressões de MMP-2.

**Métodos:**

Um total de noventa e dois pacientes com primeiro IM passaram por exames de imagens por ressonância magnética cardiovascular 3 Tesla realizados 2 semanas (linha de base) e 6 meses após o IM. Medimos o mapeamento T1 com sequências MOLLI. O VEC foi obtido após o realce pelo gadolínio. O VEC e a massa do VE foram usados para calcular o MVi e o CVi. A RA foi definida como um aumento de ≥ 12% no volume diastólico final do VE em 6 meses. As MMPs foram medidas usando-se um sistema de imunoensaio multiplex em grânulos no primeiro dia (linha de base) e 2 semanas após o IM. Um P valor <0,05 foi aceito como estatisticamente significativo.

**Resultados:**

Os níveis de linha de base de MVi média e VEC médio foram mais altos no grupo com RA em comparação com o grupo sem RA (42,9±6,4 vs. 39,3±8,2 %, p= 0,037; 65,2±13,7 vs. 56,7±14,7 mL/m^2^, p=0,010; respectivamente). Os níveis de CVi eram semelhantes entre os grupos. Foi encontrada uma correlação positiva entre os níveis de linha de base de MMP-2 e os níveis de linha de base de VEC (r=0,535, p<0,001) e MVi (r=0,549, p<0,001). O aumento dos níveis de ΔMVi foi um preditor independente da RA (RC=1,03, p=0,010). O ΔMVi teve um desempenho diagnóstico superior quando comparado ao ΔVEC na previsão do (ΔAUC: 0,215±0,07, p<0,001).

**Conclusão:**

Níveis altos de MVi estão associados à RA, e o ΔMVi foi um preditor independente de RA. Isso pode estar associado à liberação de MMP-2 devido ao aumento da resposta inflamatória.

## Introdução

O infarto agudo do miocárdio (IM) inicia uma resposta inflamatória envolvendo a interação da matriz extracelular (MEC) e a ativação neuro-humoral e, depois disso, avança com aumento de fibroblastos.^1^ Fibroblastos produzem as proteínas estruturais da MEC e podem causar tempestades de citocina e produção excessiva de matriz metaloproteinases (MMPs) em respostas inflamatórias extremas.^2^ Esses fatores contribuem para a produção e o acúmulo das proteínas de MEC em excesso, causando um efeito mal adaptativo nas propriedades estruturais e funcionais do coração e resultando em remodelação adversa (RA) do ventrículo esquerdo (VE).^3^

O processo de desenvolvimento da RA está associado à expansão da matriz intersticial e alterações dinâmicas na rede da MEC.^4^ O espaço extracelular aumenta quando o miocárdio saudável é substituído por fibrose ou tecido cicatricial.^5^ O aumento da MEC é convertido em valores quantitativos por meio dos valores de T1 e da fração do volume extracelular (VEC) avaliados pelo mapeamento T1 e por imagens por ressonância magnética cardiovascular (IRMC).^6^ Além disso, os índices derivados do VEC e do volume miocárdico (volume de matriz do VE e volume celular) permitem a avaliação da reversibilidade das alterações em compartimentos celulares e extracelulares.^7^ As MMPs, que são enzimas proteolíticas zinco-dependentes, têm um papel significativo na modulação da MEC e, portanto, têm significância prognóstica na remodelação do VE.^8^ Entretanto, não conseguimos encontrar nenhum estudo prévio avaliando a contribuição de alterações nos compartimentos celular e extracelular para o desenvolvimento de RA após IM e a relação dessas alterações e as MMPs. Portanto, neste estudo, o papel prognóstico (Δ) do mapeamento T1, incluindo o volume de matriz do VE e volume celular com desenvolvimento de RA em pacientes com primeiro IM com supradesnivelamento do segmento ST (IAMCSST) e sua relação com a MMP-2 foram investigados.

## Materiais e métodos

### População do estud

A pesquisa foi realizada entre junho de 2015 e junho de 2018 como estudo prospectivo multicêntrico, de acordo com a Declaração de Helsinki, e foi aprovada pelo comitê de ética local (data/nº da decisão: 24.06.2013/106). O termo de consentimento por escrito foi obtido de todos os pacientes. Com base em estudos anteriores, a taxa de desenvolvimento de RA no 6º mês de acompanhamento após o IM foi considerada em 30% e o tamanho de amostra estimado foi de, no mínimo, 46 pacientes, com um valor alfa de 0,05 e poder de 0,80.

**Figura 1:**
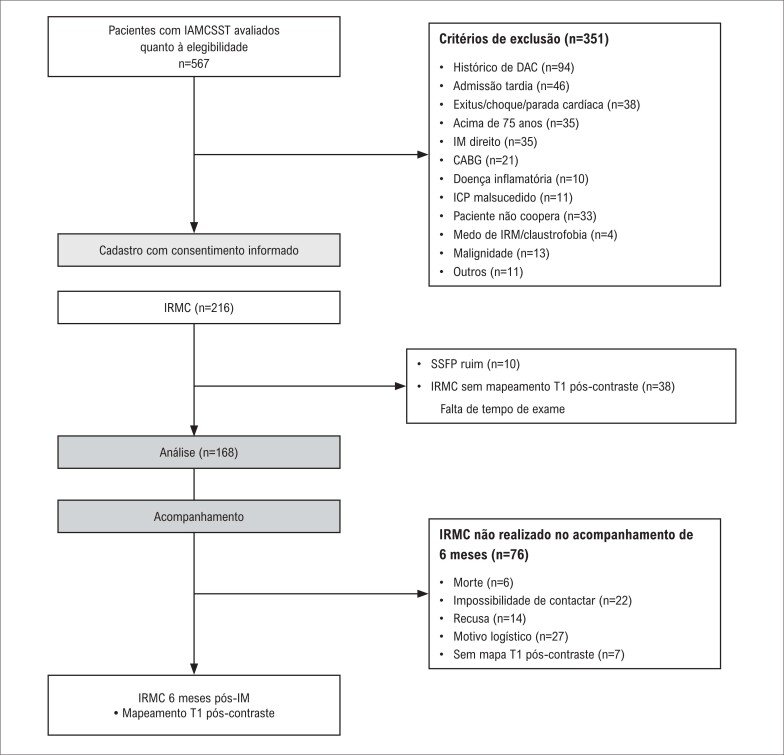
Fluxograma do estudo de coorte. IRMC: imagens por ressonância magnética cardíaca; SSFP: precessão livre no estado estacionário; IAMCSST: IM com supradesnivelamento do segmento ST; IM: infarto do miocárdio; DAC: doença arterial coronariana; CABG: enxerto de bypass de artéria coronária; ICP: Intervenção coronária percutânea.

Foram avaliados 567 pacientes acima de 18 anos de idade que foram admitidos na emergência com diagnóstico de IAMCSST pela primeira vez e que passaram por intervenção coronária percutânea (ICPp). Detectou-se que 351 pacientes não atendiam aos critérios de inclusão e foram excluídos do estudo. Foram incluídos no estudo noventa e dois pacientes que passaram por ICPp nas 12 horas após o início da dor torácica e cujo mapeamento T1 foi avaliado por IRMC no 6º mês de acompanhamento após o IM (Figura 1). O diagnóstico de IAMCSST foi feito de acordo com a terceira definição universal de IM^9^ e o tratamento foi planejado de acordo com as diretrizes da Sociedade Europeia de Cardiologia (ESC) atualizadas mais recentemente.^10^

Os critérios de exclusão do estudo foram histórico anterior de doença arterial coronariana, admissão hospitalar tardia (>12 horas), choque cardiogênico (pressão arterial sistólica de ≤90 mmHg), necessidade de suporte hemodinâmico, histórico anterior de isquemia/infarto silencioso, doença inflamatória sistêmica ou doença autoimune, uso crônico de corticosteroide ou medicamento anti-inflamatório, gravidez/parto/amamentação nos últimos 3 meses, novo infarto do miocárdio após enxerto de bypass na artéria coronária emergencial ou eletivo depois de angiografia, ICPp malsucedido, medo de IRM e claustrofobia.

### Protocolo do estudo

Todos os dados pertinentes foram registrados nos prontuários dos pacientes à medida que foram obtidos durante o acompanhamento, incluindo dados demográficos e dados sobre resultados clínicos, laboratoriais e radiológicos. O cálculo do escore do *Global Registry of Acute Cardiac Events Risk* - GRACE (Registro global do risco de eventos cardíacos agudos) foi utilizado com a calculadora oficial (www.gracescore.org). Durante o acompanhamento, os exames de IRMC foram realizados em todos os pacientes participantes 2 semanas (linha de base) e 6 meses após o IAMCSST usando os mesmos dispositivos (scanner Magnetom Skyra 3-T, Siemens Medical Systems, Erlangen, Alemanha) em todos os centros participantes. Os dados obtidos assim foram coletados para serem avaliados por um indivíduo com experiência considerável em interpretações de resultados de IRMC. As interpretações foram cegas, já que o indivíduo que as realizou não tinha conhecimento de dados específicos do paciente ou dos resultados relevantes. Para avaliar as MMPs, foram realizadas avaliações nesses pacientes no primeiro dia (linha de base) e 2 semanas após o IAMCSST. Os soros foram mantidos a uma temperatura de -80 °C até serem testados. Após a coleta do soro de amostras completas, os parâmetros relevantes foram quantificados pela mesma equipe de laboratório, usando o mesmo dispositivo em uma sessão única no Laboratório de tipagem histológica e Centro de diagnóstico genético do hospital relevante.

### Exames laboratoriais

Amostras de sangue venoso foram obtidas no momento da admissão e centrifugadas a 1500 rpm por 10 minutos, e um hemograma completo (HMG) e parâmetros bioquímicos foram analisados. Os parâmetros do HMG foram medidos com um analisador hematológico Sysmex XN-1000 (Sysmex Corporation, Kobe, Japão) e as medições de hemoglobina foram realizadas pelo método fotométrico. O colesterol total foi medido pelo método enzimático colorimétrico homogêneo (autoanalisador Hitachi Modular P800, Roche Diagnostics Corp., Indianapolis, IN, EUA) e os níveis de colesterol de lipoproteína de baixa densidade (LDL) foram determinados pelo método de Friedewald.^11^ Os níveis de troponina I cardíaca sérica (cTn-I) foram medidos em um analisador Dimension (Dade Behring Diagnostics, Amersfoort, Holanda) com um método de imunoensaio enzimático de uma etapa baseado no princípio de sanduíche.

As medições de MMP-2 foram repetidas duas vezes. Soros previamente congelados foram descongelados em gelo e os valores de MMP-2 foram analisados em seguida com o auxílio de um sistema de imunoensaio multiplex em grânulos (Bio-Plex Pro Human Inflammation Panel, Bio-Rad Laboratories, Hercules, CA, EUA). Para medir e quantificar o desenvolvimento de imunocomplexos de sanduíche selecionados, o sistema Bio-Plex MAGPIX System (Bio-Rad) foi aplicado aos conjuntos de grânulos relevantes. As concentrações finais de analitos foram determinadas com o auxílio do software Bio-Plex Manager v.5.0 (Bio-Rad). Amostras de sangue foram coletadas em momentos semelhantes para evitar o efeito do ritmo diário nas diferenças de expressão de marcadores inflamatórios. Portanto, 46 pacientes que tiveram suas coletas no período da manhã (8:00-12:00) foram examinados quanto a expressões de MMP-2.

### Imagens por ressonância magnética cardíaca

No processo de obtenção de dados de IRMC, foi obtida uma única vista com 4 câmaras e seções de cine de eixo curto (espessura do segmento de 6 mm em intervalos de 10 mm), bem como uma única vista com 2 câmaras. Foram realizadas avaliações dos índices de função sistólica do VE com a aplicação de sequências de pulso rápido (turbo-FLASH) de eletrocardiograma retrospectivo com tempo de eco (TE) de 1,42 ms, tempo de repetição (TR) de 39 ms, ângulo de giro de 57° e dimensão do voxel de 1,67 × 1,67 × 6 mm. Os dados de IRMC obtidos dessa forma em seguida foram transferidos inteiramente para uma estação de trabalho. A partir daí, o volume sistólico final (VSF) do VE e o volume diastólico final (VDF) do VE foram determinados por um leitor, usando o software de imagem Siemens syngo.via VA30. Nesse processo, as bordas endoteliais das fases sistólica final e diastólica final de imagens de eixo curto, que incluíram o VE dentro de uma faixa de espaço da linha anular da mitral até o vértice, foram traçadas manualmente com exceção dos músculos papilares. Para imagens cine, a primeira fase foi considerada a fase diastólica final, e a fase sistólica final foi identificada visualmente com base na interrupção dos movimentos do VE para o interior.^12^

A dimensão do infarto foi calculada pela soma do volume de hiper-realce por segmento, e apresentado como porcentagem da massa total do VE.

A definição de RA foi aplicada à luz dos valores limítrofes de VDF-VE amplamente aceitos (∆VDF-VE >12%).^13^

### Mapa T1

Mapas T1 foram obtidos antes e 15 minutos depois do processamento do contraste por gadolínio. Três segmentos axiais curtos (apical, médio e basal) foram considerados ao se obter o mapeamento T1 com aplicação de uma sequência de protótipo investigativo de recuperação de inversão de *look-locker* modificada (MOLLI) (Siemens Healthcare, Malvern, PA, EUA),^14,15^ juntamente com a incorporação de um algoritmo de registro automático, conforme destalhado em uma publicação anterior.^16^ No processo de aquisição de dados cardíacos de MOLLI T1, um total de 3 experimentos *look-locker* preparados para recuperação de inversão foram realizados de acordo com um único protocolo.^14^ Para as etapas descritas aqui, os parâmetros de IRMC aplicados incluíram uma largura de banda de ~1090 Hz/pixel, ângulo de giro de 35°, tempo de eco (TE) de 1,1 ms, T1 experimental inicial de 100 ms, incremento de TI de 80 ms, matriz de 192 × 124 pixels, resolução espacial de 2,2 × 1,8 × 8,0 mm, espessura do segmento de 8 mm, e tempo de digitalização de 17 batimentos cardíacos.

### Medição do VEC

Regiões de interesse (ROI) foram obtidas do miocárdio remoto em uma localização a 180° da zona de infarto, da zona de infarto através de toda a área da lesão registrada, e do reservatório de sangue do VE. Essas ROI especificadas foram copiadas com a aplicação de mapas T1 antes e depois da administração de um agente de contraste. Nesse processo também foram aplicadas correções manuais para manter as margens de separação das interfaces de tecido. Os valores de VEC foram calculados com base nas razões de valores da área de infarto T1 relevantes previamente obtidas antes e depois da administração de agentes de contraste em cada ROI. Consequentemente, não foi necessário fazer registros entre mapas T1 para se obter os cálculos de VEC com precisão. A equação (1) foi usada aqui para VEC, em que λ = ΔR1_miocárdio_/ΔR1_sangue_, ΔR1 = R1_pós-contraste_ – R1_pré-contraste_, e R1 = 1/T1. O hematócrito (HCT) também foi avaliado enquanto esses exames estavam sendo realizados.


(1) VEC=(1−HCT)×λ

**Figura 2:**
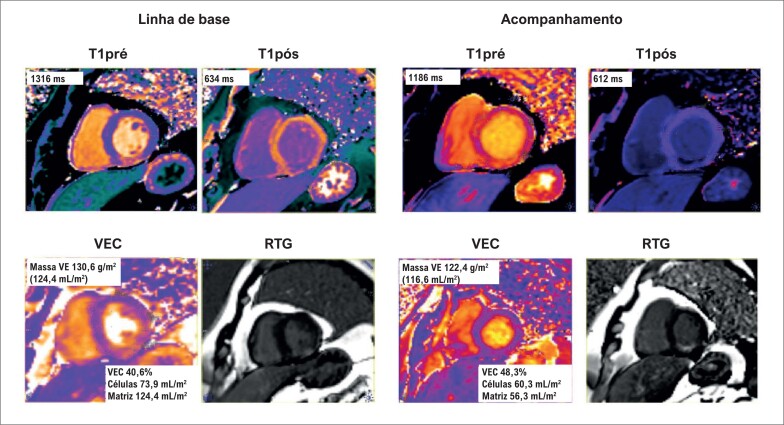
Mapas de T1 e valores de VEC após períodos de IM. CVi: índice de volume celular; VEC: volume extracelular; MVi: índice de volume de matriz; VE: ventrículo esquerdo.

O índice de volume de matriz foi calculado pelo produto do volume miocárdico do VE (massa do VE dividida pela gravidade específica do miocárdio [1,05 g/mL]) e VEC ou (1 – VEC) para índice de volume celular.^17^ Exemplos dessas medições de mapas T1, e VEC e compartimentos são apresentados na Figura 2.

### Análise estatística

As variáveis categóricas foram apresentadas como números e porcentagens, e as comparações entre os grupos foram realizadas usando-se os testes qui-quadrado, correção de Yates e Fisher. A distribuição normal das variáveis numéricas foi avaliada com os testes de Kolmogorov-Smirnov e os resultados com distribuição normal foram mostrados como média ± desvio padrão, enquanto os com distribuição não normal foram apresentados como mediana (faixa interquartil (FIQ)). Comparações entre os grupos de variáveis numéricas foram realizadas com os testes T de Student ou U de Mann-Whitney. A alteração nos parâmetros de IRMC entre 2 semanas e 6 meses foi avaliada com o teste T pareado ou o teste de Wilcoxon de acordo com a normalidade da distribuição. Para comparar os parâmetros de IRMC considerados, um modelo misto de medidas repetidas (MMRM) foi definido com o objetivo de comparar parâmetros de IRMC e níveis de MMP-2 entre os grupos no período pós-IM. Essa correlação entre variáveis numéricas foi testada pela análise de correlação de Spearman. Os efeitos na RA foram avaliados pela realização da análise de regressão logística univariada. Fatores de risco em potencial associados à RA (p<0,25) foram incluídos nesses modelos de regressão logística multivariada.^18,19^ Nos modelos de regressão multivariada stepwise, fatores de risco potenciais e alterações nos parâmetros de IRMC de 2 semanas a 6 meses após o IM foram incluídos. A análise da curva de característica de operação do receptor (ROC) foi realizada para estabelecer uma discriminação diagnóstica dos parâmetros de mapeamento T1 da RA. O software IBM SPSS Statistics (IBM Corp., Armonk, NY, EUA) foi usado para todas as análises e p<0,05 (*) foi aceito como estatisticamente significativo.

## Resultados

Foram avaliados 567 pacientes que foram admitidos na emergência com diagnóstico de IAMCSST (média de idade: 56,7±15,2 anos, 14,3% do sexo feminino). Foram incluídos na análise noventa e dois pacientes que atenderam aos critérios de exclusão e que foram avaliados por IRMC (média de idade: 54,1±9,0 anos). A maioria dos pacientes era do sexo masculino (90,2%) com um perfil de risco representativo de doença cardiovascular. As características demográficas, clínicas e de IRMC são apresentadas nas Tabelas 1 e 2. A RA foi detectada em 32,6% (n=30) de todos os pacientes 6 meses após o IM. A troponina I cardíaca mediana e os níveis medianos de proteína C reativa de alta sensibilidade foram mais altos no grupo com RA em comparação com o grupo sem RA. Não houve diferença significativa entre as características demográficas e outras características clínicas dos pacientes dos grupos com e sem RA (Tabela 1).

**Tabela 1:** Achados demográficos e laboratoriais

Variáveis			Total população n=92	Remodelação adversa		p
				Sim n=30	Não n=62	
**Achados demográficos**						
	Idade, anos		54,1±9,0	53,4±8,5	54,4±9,3	0,623
	Sexo masculino, n(%)		83(90,2)	25(83,3)	58(93,5)	0,241
	IMC, kg/m^2^		26,7±4,3	27,0±3,7	26,5±4,6	0,704
	ASC, m^2^		1,9±0,2	1,9±0,2	1,9±0,2	0,997
	Hipertensão, n (%)		40(43,5)	12(40,0)	28(45,2)	0,661
	Diabetes, n (%)		27(29,3)	9(30,0)	18(29,0)	0,999
	Dislipidemia, n (%)		24(26,1)	10(33,3)	14(22,6)	0,315
	Tabagismo, n (%)		46(50,0)	19(63,3)	27(43,5)	0,113
**Achados clínicos**						
	Frequência cardíaca, bpm		76,9±16,8	75,2±12,6	77,8±18,7	0,509
	PAS, mm Hg		123±15,5	124,1±14,7	122,4±16,1	0,657
	PAD, mm Hg		77,2±12,2	77,8±11,2	77,0±12,8	0,785
	Tempo sintoma-balão, min		312,2±68,4	304,6±67,2	317,4±69,8	0,535
	Tempo porta-balão, min		28,1±8,8	27,2±8,4	29,5±9,0	0,358
	ARI, n(%)					
		ADAE	67(72,8)	22(73,3)	45(72,6)	0,999
		Cx	25(27,2)	8(26,7)	17(27,4)	
	Escore GRACE		128,5±30,4	131,1±24,0	127,3±33,2	0,529
	Fluxo TIMI pré-ICP, n(%)					
		0	54(58,7)	18(60,0)	36(58,1)	0,848
		1	15(16,3)	5(16,7)	10(16,1)	
		2	2(17,5)	5(16,7)	12(19,4)	
		3	6(6,5)	2(6,7)	4(6,5)	
	Fluxo TIMI pós-ICP >2, n(%)		90(97,8)	29(96,7)	61(98,4)	0,999
**Achados laboratoriais**						
	cTn-I, ng/L		46,4(37,7-57,8)	56,5(50,4-60,0)	41,7(35,5-48,0)	<0,001
	Hemoglobina, g/dL		13,8±1,5	14,0±1,8	13,6±1,4	0,375
	WBC, x10^9^/L		12,3±3,3	12,4±3,2	12,2±3,4	0,829
	Linfócitos, x10^9^/L		2,3±0,8	2,2±0,8	2,4±0,8	0,149
	Neutrófilos, x10^9^/L		8,4±2,1	8,8±1,9	8,2±2,2	0,190
	Monócitos, x10^9^/L		0,7±0,2	0,8±0,2	0,7±0,2	0,884
	Plaquetas, x10^9^/L		301,7±60,7	318,6±54,0	293,5±62,5	0,062
	Glicemia, mg/dL		112(75-140)	114(100-149)	107(83-139)	0,390
	Colesterol total, mg/dL		197(160-220)	191(155-211)	200(164-240)	0,264
	LDL, mg/dL		132(100-157)	119(101-144)	136(100-170)	0,261
	HDL, mg/dL		41,3±9,2	41,7±9,5	41,1±9,1	0,781
	Proteína C reativa, mg/L		24,2(13-31,3)	28,0(16-41,2)	18,3(11,7-26,3)	0,024
**Tratamento após a alta, n(%)**						
	ECA/BRA		90(97,8)	30(100,0)	60(96,8)	0,816
	Betabloqueadores		90(97,8)	29(96,7)	61(98,4)	0,999
	Estatinas		91(98,9)	30(100,0)	61(98,4)	0,999

Variáveis numéricas são mostradas como média ± desvio padrão ou mediana (FIQ). Variáveis categóricas são expressas como números (%). ASC: área de superfície corporal; ECA: enzima conversora da angiotensina; BRA: bloqueador do receptor da angiotensina II; IMC: índice de massa corporal; Cx: artéria circunflexa; PAD: pressão arterial diastólica; HDL: lipoproteína de alta densidade; ARI: artéria relacionada ao infarto; ADAE: artéria descendente anterior esquerda; LDL: lipoproteína de baixa densidade; ICP: intervenção coronária percutânea; PAS: pressão arterial sistólica; TIMI: trombólise no infarto do miocárdio.

**Tabela 2:** Resultados da RMC no momento agudo e acompanhamento

Variáveis			Total população n=92	Remodelação adversa		p
				Sim n=30	Não n=62	
**Segunda semana**						
	FEVE, %		46,8±9,6	46,5±9,6	47,0±9,6	0,818
	VDF-VE, mL		155(130,1-172,5)	153(135-176,7)	157,6(129-170)	0,723
	VSF-VE, mL		83,4(60,1-112,5)	93,5(70,7-128)	78,3(60-102)	0,207
	MiVE, g/m^2^		144(130-165)	147(133-176)	143(128-162)	0,257
	Dimensão do infarto, % do VE		15(11-22)	18(12-21)	15(10-21)	0,407
	T1 nativo, ms					
		Pré-contraste	1411,0±148,8	1421,2±162,8	1406,5±142,8	0,692
		Pós-contraste	490,8±88,2	493,5±90,7	489,5±87,7	0,837
	VEC, %		40,1±7,4	42,9±6,4	39,3±8,2	0,037
	MVi, mL/m^2^		59,5±14,9	65,2±13,7	56,7±14,7	0,010
	CVi, mL/m^2^		88,0±15,0	86,3±13,6	88,9±15,6	0,447
**Seis meses**						
	FEVE, %		47,7±9,7	42,9±10,3	50,0±8,5	0,001
	VDF-VE, mL		155,4(130-180,9)	180,7(159-227)	140(125,6-162,3)	<0,001
	VSF-VE, mL		79(59,7-116,1)	115,6(80-164)	68,9(54,4-90,7)	<0,001
	MiVE, g/m^2^		126(116-144)	138(122-166)	123(112-137)	0,002
	Dimensão do infarto, % do VE		12(8-16)	15(10-18)	11(7-15)	0,035
	T1 nativo, ms					
		Pré-contraste	1309,4±135,7	1325,2±117,0	1302,4±136,7	0,490
		Pós-contraste	455,3±82,1	438,7±69,4	463,3±86,9	0,179
	VEC, %		45,8±6,2	49,7±6,1	44,0±5,4	<0,001
	MVi, mL/m^2^		60,5±15,2	70,7±12,1	55,6±11,8	<0,001
	CVi, mL/m^2^		70,6±11,0	70,9±12,2	70,4±10,4	0,851

Variáveis numéricas são mostradas como média ± desvio padrão e mediana (FIQ). CVi: índice de volume celular; VEC: volume extracelular; VDF-VE: volume diastólico final do ventrículo esquerdo; FEVE: fração de ejeção ventricular esquerda; VSF-VE: volume sistólico final do ventrículo esquerdo; MiVE: índice de massa do ventrículo esquerdo; MVi: índice de volume de matriz.

No período pós-IM agudo, os níveis médios de T1 do miocárdio da zona de infarto não foi significativamente diferente entre os grupos com e sem RA. Os níveis médios de VEC e MVi foram maiores no grupo com RA quando comparados aos do grupo sem RA. Aos 6 meses após o IM, os níveis médios de VEC e MVi eram mais altos no grupo com RA em comparação aos do grupo sem RA (Tabela 2).

As alterações dinâmicas do IRMC 6 meses após o IM foram resumidas na Tabela 3. Da mesma forma, houve uma redução semelhante nos valores de T1 nativos no miocárdio da zona de infarto após 6 meses nos grupos com e sem RA, enquanto houve um aumento maior nos níveis de VEC no grupo com RA. Os níveis de MVi aumentaram significativamente após 6 meses nos pacientes do grupo com RA, enquanto não houve nenhuma diferença significativa no grupo sem RA. Os níveis de CVi diminuíram significativamente nos grupos com e sem RA, e essa diminuição foi semelhante entre os grupos.

**Tabela 3:** Alterações dinâmicas no miocárdio lesionado de acordo com a presença de remodelação adversa

Variáveis	Remodelação adversa	Segunda semana	Sexto mês	p^1^	p^2^
MiVE, g/m^2^	Não	143(128-162)	123(112-137)	<0,001	0,011
	Sim	147(133-176)	138(122-166)	<0,001	
Dimensão do infarto, % do VE	Não	15(10-21)	11(7-15)	<0,001	0,715
	Sim	18(12-21)	15(10-18)	<0,001	
Infarto de T1 nativo, ms	Não	1406,5±142,8	1302,4±136,7	<0,001	0,378
	Sim	1421,2±162,8	1320,2±117	0,005	
VEC, %	Não	39,3±8,2	44,0±5,4	<0,001	0,027
	Sim	42,9±6,4	49,7±6,1	<0,001	
MVi, mL/m^2^	Não	56,7±14,7	55,6±11,8	0,480	0,007
	Sim	65,2±13,7	70,7±12,1	<0,001	
CVi, mL/m^2^	Não	88,9±15,6	70,4±10,4	<0,001	0,164
	Sim	86,3±13,6	70,9±12,2	<0,001	

Variáveis numéricas são mostradas como média ± desvio padrão ou mediana (FIQ).

1 p Segunda semana vs. sexto mês dentro dos grupos com remodelação.

2 p Comparação das alterações no acompanhamento (grupos com remodelação adversa: Não vs. Sim). CVi: índice de volume celular; VEC: volume extracelular; MiVE: índice de massa do ventrículo esquerdo; MVi: índice de volume de matriz.

**Figura 3:**
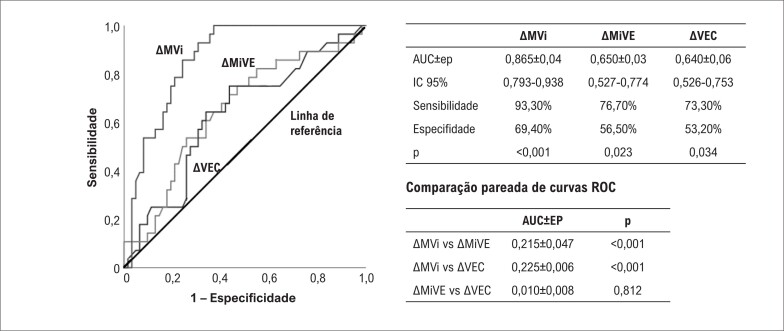
Desempenho diagnóstico de ΔVEC, ΔMiVE e ΔMVi na previsão da RA. AUC: área sob a curva; ΔAUC: diferença da área sob a curva; IC: intervalo de confiança; VEC: volume extracelular; MiVE: índice de massa do ventrículo esquerdo; MVi: índice de volume de matriz; EP: erro padrão.

No modelo de regressão I que examina a relação entre RA e alterações dinâmicas 6 meses após o IM, níveis aumentados de ΔMiVE e de ΔVEC foram preditores independentes de RA. No modelo de regressão II, MVi e CVi derivados deles foram adicionados em vez de MiVE e VEC. Da mesma forma, um aumento dos níveis de ΔMVi foi um preditor independente da RA. O modelo II teve um desempenho mais alto explicando a possibilidade de RA comparado ao modelo I (modelo I: Nagelkerke R^2^=0,537 vs. modelo II: Nagelkerke R^2^=0,615) (Tabela 4). Além disso, ΔMVi teve um desempenho diagnóstico superior comparado a ΔVEC e ΔMiVE para prever a RA (Figura 3).

**Tabela 4:** Associações multivariadas de parâmetros de mapeamento T1 com remodelação adversa no sexto mês após o IM

Variáveis		Univariada			Multivariada		
		RC	IC 95%	p	RC	IC 95%	p
**Modelo I**							
	cTn-I	1,05	1,01-1,09	0,011	1,28	1,05-1,55	0,013
	Proteína C reativa	1,07	1,01-1,12	0,017	1,15	1,01-1,32	0,044
	ΔMiVE	1,28	1,12-1,48	0,010	1,36	1,14-1,78	0,012
	ΔDimensão do infarto	1,02	0,98-1,06	0,249	-	-	-
	ΔInfarto de T1 nativo	1,01	0,97-1,06	0,154	-	-	-
	ΔVEC	1,04	1,01-1,08	0,025	1,05	1,02-1,09	0,041
					Nagelkerke R^2^=0,537, p<0,001		
**Modelo II**							
	cTn-I	1,05	1,01-1,09	0,011	1,32	1,12-1,56	0,014
	Proteína C reativa	1,07	1,01-1,12	0,017	1,15	1,03-1,30	0,020
	ΔDimensão do infarto	1,02	0,98-1,06	0,249	-	-	-
	ΔInfarto de T1 nativo	1,01	0,97-1,06	0,154	-	-	-
	ΔMVi	1,06	1,01-1,11	0,004	1,03	1,01-1,06	0,010
	ΔCVi	0,97	0,95-0,99	0,089	-	-	-
					Nagelkerke R^2^=0,615, p<0,001		

Fator de confusão, incluindo parâmetros de idade, sexo masculino, tabagismo, linfócitos, neutrófilos e plaquetas, foram ajustados em todas as análises. Δ: alteração nos parâmetros de mapeamento T1 de 2 semanas a 6 meses após o IM. IC: intervalo de confiança; CVi: índice de volume celular; VEC: volume extracelular; MiVE: índice de massa do ventrículo esquerdo; MVi: índice de volume de matriz; RC: razão de chance.

**Figura 4:**
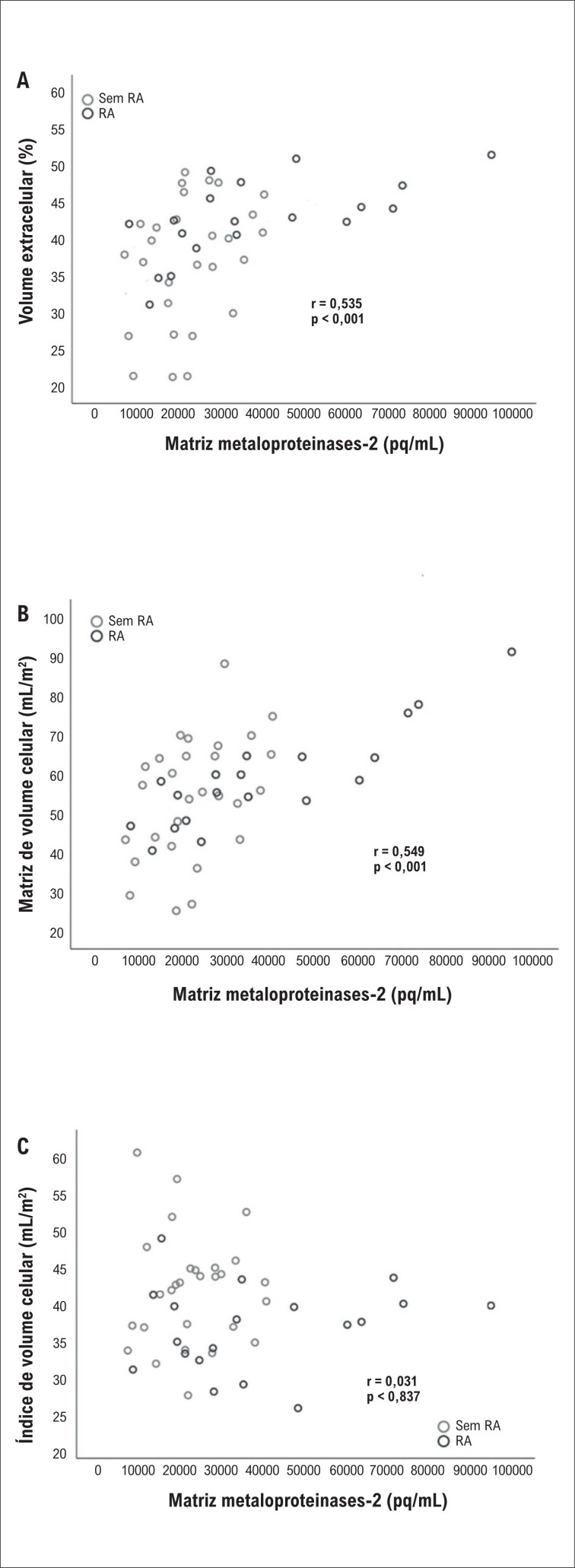
Relação entre matriz metaloproteinases-2 e parâmetros VEC (A), MiV (B) e CVi (C). RA: remodelação cardíaca adversa; CVi: índice de volume celular; VEC: volume extracelular; MVi: índice de volume de matriz.

Os níveis medianos de MMP-2 no primeiro dia após o IM eram mais alto no grupo com RA comparado ao grupo sem RA [33241,6 (FIQ: 18811,3-60196,5) vs. 21333 (FIQ: 16043,3-28784,3) pq/mL, p=0,026], enquanto não houve diferença significativa 2 semanas após o IM [32811,3 (FIQ: 19906,7-51487,2) vs. 25572,8 (FIQ: 16831-46611,6) pq/mL, p=0,340]. Os níveis medianos de MMP-2 não eram significativamente diferentes depois de 2 semanas em comparação com o primeiro dia após o IM no grupo com RA (33241,6 vs. 32811,3 pq/mL, p=0,809), mas aumentaram no grupo sem RA (21333 vs. 25572 pq/mL, p=0,046). Foi encontrada uma correlação positiva entre os níveis de linha de base de MMP-2 e os níveis de linha de base de MiVE, VEC e MVi (Figura 4) (Tabela 5).

**Tabela 5:** Relação entre matriz metaloproteinases-2 e parâmetros de RMC

Variáveis		Primeiro dia após o IM MMP-2		2 semanas após o IM MMP-2	
		r	p	r	p
**2 semanas**					
	MiVE	0,301	0,047	0,089	0,551
	Dimensão do infarto	0,024	0,878	0,221	0,144
	Infarto de T1 nativo	0,135	0,367	0,166	0,263
	VEC (%)	0,535	<0,001	0,355	0,014
	MVi	0,549	<0,001	0,325	0,029
	CVi	0,031	0,837	0,143	0,338

CVi: índice de volume celular; VEC: volume extracelular; MiVE: índice de massa do ventrículo esquerdo; MMP-2: matriz metaloproteinases-2; MVi: índice de volume de matriz.

## Discussão

Os principais achados deste estudo foram que, em pacientes com IAMCSST que desenvolveram RA durante o acompanhamento de 6 meses: 1) Os valores de VEC aumentaram mais proeminentemente após 6 meses; 2) Esse aumento foi na direção do volume da matriz; 3) Foi identificada uma correlação positiva entre os níveis de MMP-2 e os níveis de VEC e MVi; 4) ΔMVi no acompanhamento de 6 meses foi superior a ΔVEC na previsão da RA; e 5) O modelo de regressão em que o MVi foi incluído foi superior para explicar RA.

O mapa T1 quantitativo mede o tempo de relaxamento T1 baseado em pixels no miocárdio. O tempo de relaxamento T1 varia dependendo da diferenciação ao redor do tecido e reflete processos patológicos no nível do tecido.^20^ Em pacientes com insuficiência cardíaca, relatou-se que os tempos de relaxamento de T1 têm correlação positiva com a fibrose detectada por biópsia.^17^ Quantidades aumentadas de fibrose miocárdica atrapalham a estrutura do miocárdio e causam disfunção diastólica e sistólica.^21^ Isso é caracterizado pelo acúmulo excessivo de proteínas MEC. Aumentos no total de água, edema e depósito de colágeno no miocárdio se devem ao resultado da resposta inflamatória em valores de T1 nativo.^22^ Diminuições nos valores de T1 nativo em pacientes de IAMCSST, independentemente do desenvolvimento de RA podem ser associadas à preservação da capacidade de cura, reabsorção de edema, e tecido necrótico no miocárdio infartado. A fibrose segmentar se desenvolve na área da necrose e a fibrose intersticial ou substitutiva se desenvolve em áreas não necróticas após o IM. Valores altos de VEC refletem depósitos excessivos de colágeno, cicatrização, e fibrose intersticial extensa^5^ e podem ser indicadores importantes de RA.^23^

Isso é consistente com o fato de que VEC foi um preditor independente no modelo de regressão I estabelecido na presente pesquisa. Além disso, a eliminação do T1 nativo corrobora a ideia de que o espaço extracelular tem um papel mais prognóstico no desenvolvimento de RA.^22,24^

O mapa T1 introduziu um novo conceito na prática cardiológica permitindo que o miocárdio se separe em seu compartimento celular (principalmente miócitos) e compartimento intersticial (principalmente colágeno ou edema). O VEC reflete uma razão de volume relativa do volume miocárdico total. Entretanto, ele não reflete sensivelmente as alterações dinâmicas no tecido miocárdico quando há alteração dos componentes celular e extracelular.^17^ Portanto, a avaliação do VEC dividindo-o em índices de matriz e volume celular pode oferecer informações mais detalhadas sobre o mecanismo da RA e fazer com que o novo paradigma de vulnerabilidade cardíaca seja mais fácil de entender e aplicar. Em estudos anteriores de doenças cardíacas diferentes, foram estabelecidos modelos de matriz e célula aumentada, foi detectada uma diminuição em seus níveis com tratamento médico de acordo com as diretrizes relevantes, e a redução foi associada à melhoria no volume e nas funções cardíacas.^7,17^ Em nosso estudo, embora o tempo sintoma-balão não tenha sido diferente entre os pacientes que desenvolveram e os que não desenvolveram RA após o IM, todos os pacientes receberam o tratamento de diretriz e foi detectada uma redução significativa no índice de volume celular. Além disso, não houve diferença significativa na dimensão do infarto na segunda semana entre os dois grupos de estudo. Entretanto, uma redução semelhante em CVi foi observada entre os que desenvolveram RA apesar de receber tratamento semelhante, mas concluiu-se que a alta variação incremental no MVi foi um preditor independente de RA. Esse achado é consistente com os mecanismos pelos quais a recuperação de miócitos pode preceder a remodelação adaptativa no componente extracelular.^7,25^ Além disso, pode indicar que os volumes celulares se normalizam mais cedo que os volumes de matriz. Por outro lado, níveis altos de MMPs podem contribuir para a normalização tardia de volumes de matriz, que pode estar associada a uma melhoria posterior na dimensão do infarto após 6 meses em pacientes com RA.^26^

O modelo de regressão incluindo o VEC demonstrou desempenho diagnóstico menor comparado ao modelo de regressão, incluindo componentes do VEC. Por outro lado, o desempenho diagnóstico mais baixo do VEC comparado a componentes celulares e extracelulares na análise ROC corrobora o fato de que o VEC pode ser menos sensível a alterações histológicas. O desempenho diagnóstico superior do MVi para prever a RA, comparado ao VEC e ao CVi, pode indicar que ele pode ser mais sensível a alterações histológicas. Todas as constatações apresentadas aqui destacam a importância dos fibroblastos cardíacos nas alterações da estrutura dinâmica da matriz extracelular, incluindo a fibrose difusa (volume de matriz).^27^ A associação entre fibroblastos cardíacos e alterações no ciclo de colágeno destaca a natureza dinâmica da matriz extracelular. A quantificação de MVi poderia acrescentar mais informações preditivas e corroborar a reversibilidade do desenvolvimento da RA, especialmente considerando sua associação a MMPs. Na prática clínica, o MVi pode oferecer informações detalhadas sobre o mecanismo de RA refletindo melhor as alterações no tecido miocárdico. Portanto, o MVi pode ser um guia em termos de prognóstico desfavorável após o IAMCSST. Também pode ser importante em termos de desfechos,^17^ porque a fibrose focal e a fibrose extensa demonstraram ser preditores univariados de resultado.^28^ Pesquisas anteriores demonstraram que fibroblastos cardíacos constituem 60 a 70% de todas as células miocárdicas, exercendo uma influência crucial no processo de reparo miocárdico para garantir a continuação das funções cardíacas pós-lesão. Perfis pró-inflamatórios e pró-fibróticos realçados podem levar a aumentos graduais na rigidez o miocárdio bem como uma conformidade miocárdica diminuída juntamente como disfunção diastólica e sistólica ventricular. Esse processo está fundamentado na ativação precoce de MMPs.^29,30^ MMP-2 podem ser autoativadas na MEC pela ação de radicais livres produzidos por macrófagos teciduais ativados.^31^ Uma resposta inflamatória excessiva pode resultar em produção excessiva de MMP-2, e isso, por sua vez, pode ter um papel na RA causando vulnerabilidade cardíaca.^32^ O mecanismo desse processo pode ser orientado para um aumento de VEC (principalmente com o aumento do volume de matriz) resultante de uma produção de MMP-2 excessiva devido a uma resposta inflamatória excessiva. Portanto, a ativação de MMP pode representar um alvo terapêutico viável para a regulação da transformação de MEC durante o processo patológico de desenvolvimento de RA após IM.

Este estudo, que representa uma coorte de pacientes que passaram por IAMCSST pela primeira vez, tem certas limitações. Embora os achados desta pequena amostra estejam alinhados à literatura, uma amostra maior poderia oferecer resultados mais consistentes. Por outro lado, a fisiopatologia da RA é complexa. Alterações no miocárdio da zona remota, micro RNAs e citocinas podem desempenhar papéis importantes que não puderam ser avaliados neste estudo. A consideração desses fatores em estudos futuros poderia iluminar melhor a associação entre mudança nos compartimentos do VEC com inflamação.

## Conclusão

Este estudo oferece maiores evidências para a significância fisiopatológica das características histológicas e da remodelação do VE no início do IAMCSST. Concluímos que alteração no volume de matriz, superior à alteração de VEC, após o período do IM agudo, é um preditor independente de RA, refletindo fibrose intersticial aumentada. Níveis aumentados de MMP-2 no início do IM agudo desempenham um papel importante na alteração do volume de matriz, e, portanto, podem ser um alvo terapêutico.
