# Supplementary material for: A Relação entre Compartimentos de Volume Extracelular e Matriz Metaloproteinase 2 na Remodelação do Ventrículo Esquerdo após o Infarto do Miocárdio
Source: Arq Bras Cardiol. 2022 Nov 23;119(6):946–57. [Article in Portuguese] doi: 10.36660/abc.20220061 (PMC9814815; doi:10.36660/abc.20220061)
Supplement: Translation

# Translation: The Relationship between Extracellular Volume Compartments and Matrix Metalloproteinases-2 in Left Ventricular Remodeling after Myocardial Infarction

**Authors:** Ferhat Eyyupkoca, Nilnur Eyerci, Mehmet Sait Altintas, Mehmet Ali Felekoglu, Halil Ibrahim Biter, Siho Hidayet, Serkan Sivri, Bekir Demirtas, Omer Faruk Ates

**Affiliations:** 1 Ankara Turkey Departamento de Cardiologia, Dr. Nafiz Korez Sincan State Hospital, Ankara – Turkey; 2 Kars Turkey Departamento de Biologia Médica, Kafkas University Faculty of Medicine, Kars – Turkey; 3 Istanbul Turkey Departamento de Cardiologia, Istanbul Yedikule Chest Diseases and Thoracic Surgery Training and Research Hospital, Istanbul – Turkey; 4 Yalova Turkey Departamento de Cardiologia, Atakent Hospital, Yalova – Turkey; 5 Istanbul Turkey Departamento de Cardiologia, Istanbul Haseki Training And Research Hospital, Istanbul – Turkey; 6 Malatya Turkey Departamento de Cardiologia, Inonu University Faculty of Medicine, Malatya – Turkey; 7 Kirşehir Turkey Departamento de Cardiologia, Kirsehir State Hospital, Kirşehir – Turkey; 8 Cankiri Turkey Departamento de Cardiologia, Cankiri State Hospital, Cankiri – Turkey; 9 Sakarya Turkey Departamento de Cardiologia, Sakarya University Faculty of Medicine, Sakarya – Turkey

**Keywords:** Myocardial Infarction/metabolism, Ventricular Remodeling, Myofibroblasts/cytology, Matrix Metaloproteinase, TI Mapping/cytology

## Abstract

**Background:**

Matrix metalloproteinases (MMPs) can affect myocardial extracellular volume (ECV) and its compartments, and this can provide more detailed information about the mechanism of adverse left ventricular (LV) remodeling (AR) after acute myocardial infarction (MI).

**Objectives:**

To investigate the role of changes (Δ) in ECV compartments (matrix volume (MVi) and cell volume (CVi)) in the development of AR after MI, and their relationship with MMP-2 expressions.

**Methods:**

Ninety-two first MI patients who underwent 3 Tesla cardiovascular magnetic resonance imaging performed 2 weeks (baseline) and 6 months post-MI. We measured T1 mapping with MOLLI sequences. ECV was performed post-gadolinium enhancement. ECV and LV mass were used to calculate MVi and CVi. AR was defined as an increase of ≥ 12% in LV end-diastolic volume in 6 months. MMPs were measured using a bead-based multiplex immunoassay system at first day (baseline) and 2 weeks post-MI. P <0.05 was accepted as statistically significant.

**Results:**

Mean ECV and mean MVi baseline levels were higher in AR group compared to without AR group (42.9±6.4 vs 39.3±8.2%, p= 0.037; 65.2±13.7 vs 56.7±14.7 mL/m^2^, p=0.010; respectively). CVi levels was similar between groups. A positive correlation was found between baseline levels of MMP-2 and baseline levels of ECV (r=0.535, p<0.001) and MVi (r=0.549, p<0.001). Increased ΔMVi levels was independently predictor of AR (OR=1.03, p=0.010). ΔMVi had superior diagnostic performance compared to ΔECV in predicting AR (ΔAUC: 0.215±0.07, p<0.001).

**Conclusion:**

High MVi levels are associated with AR, and ΔMVi was independently predictor of AR. This may be associated with MMP-2 release due to increased inflammatory response.

## Introduction

Acute myocardial infarction (MI) initiates an inflammatory response involving the interaction of the extracellular matrix (ECM) and neurohumoral activation and then progresses with fibroblast increase.^1^ Fibroblasts produce the structural proteins of the ECM and can cause cytokine storms and overproduction of matrix metalloproteinases (MMPs) in extreme inflammatory responses.^2^ These factors contribute to the production and accumulation of excess ECM proteins, causing a maladaptive effect on the structural and functional properties of the heart and resulting in adverse left ventricular (LV) remodeling (AR).^3^

The developmental process of AR is associated with the expansion of the interstitial matrix and dynamic changes in the ECM network.^4^ Extracellular space increases when healthy myocardium is replaced by fibrosis or scar tissue.^5^ ECM enlargement is converted to quantitative values via T1 values and extracellular volume (ECV) fraction assessed by T1 mapping by cardiovascular magnetic resonance imaging (CMRI).^6^ In addition, indices derived from the ECV and myocardial volume (LV matrix volume and cell volume) allow the assessment of the reversibility of changes in cellular and extracellular compartments.^7^ MMPs, which are zinc-dependent proteolytic enzymes, play a major role in the modulation of the ECM and thus have prognostic significance in LV remodeling.^8^ However, we could not find any previous study evaluating the contribution of changes in cellular and extracellular compartments to the development of AR after MI and the relationship between these changes and MMPs. Therefore, in this study, the prognostic role (Δ) of T1 mapping including LV matrix volume and cell volume with AR development in patients with first acute ST-segment-elevation MI (STEMI) and its relationship with MMP-2 were investigated.

## Materials and Methods

### Study population

This research was conducted between June 2015 and June 2018 as a multicenter, prospective study in accordance with the principles of the Declaration of Helsinki and was approved by the local ethics committee (Decision Date/No: 24.06.2013/106). Written consent was obtained from all patients. Based on previous studies, the rate of AR development at the 6th month of follow-up after MI was assumed to be 30% and the estimated sample size was at least 46 patients, with an alpha value of 0.05 and 0.80 power.

A total of 567 patients over 18 years of age who were admitted to the emergency department with the diagnosis of STEMI for the first time and who underwent primary percutaneous coronary intervention (pPCI) were evaluated. It was found that 351 patients did not meet the inclusion criteria and they were excluded from the study. Ninety-two patients who underwent pPCI within 12 hours from the onset of chest pain and whose T1 mapping was evaluated by CMRI at the 6th month of follow-up after MI were included in the study (Figure 1). The diagnosis of STEMI was made according to the third universal definition for MI^9^ and treatment was planned according to the most recently updated guidelines of the European Society of Cardiology (ESC).^10^

**Figure 1:** Flow diagram of the cohort study. CMRI: cardiac magnetic resonance imaging; SSFP: steady-state free precession. STEMI: STsegment-elevation MI; CAD: coronary; CABG: coronary artery bypass grafting; MI: myocardial infarction; PCI: percutaneous coronary intervention.

Study exclusion criteria were previous history of coronary artery disease, delayed admittance (>12 hours), cardiogenic shock (systolic blood pressure of ≤90 mmHg), need for hemodynamic support, previous history of silent ischemia/infarction, systemic inflammatory disease or autoimmune disease, chronic corticosteroid or anti-inflammatory medication use, pregnancy/delivery/breastfeeding in the last 3 months, myocardial reinfarction following emergency or elective coronary artery bypass grafting after angiography, failed pPCI, fear of MRI, and claustrophobia.

### Study Protocol

All pertinent data were noted within the files of the patients as they were obtained in the course of follow-up, including demographic data and data on clinical, laboratory, and radiological results. Calculation of the Global Registry of Acute Cardiac Events Risk (GRACE) Score was used by the official calculator (www.gracescore.org). In the course of follow-up, CMRI was conducted for all participating patients at 2 weeks (baseline) and at 6 months after STEMI using the same devices (Magnetom Skyra 3-T scanner, Siemens Medical Systems, Erlangen, Germany) at all participating centers. The data thus obtained were gathered to be assessed by one individual with considerable experience in reading CMRI results; this individual was blinded to the specific patient data and all relevant outcomes. To evaluate MMPs, assessments for these patients were performed on the first day (baseline) and at 2 weeks after STEMI. Serum were held at -80 °C until they were assayed. After collecting serum from whole samples, the parameters of interest were quantified by the same laboratory staff using the same device in a single session in the Tissue Typing Laboratory and Genetic Diagnosis Center of the relevant hospital.

### Laboratory testing

Venous blood samples were taken at the time of admission and centrifuged at 1500 rpm for 10 minutes, and complete blood count (CBC) and biochemistry parameters were analyzed. CBC parameters were measured with a Sysmex XN-1000 hematology analyzer (Sysmex Corporation, Kobe, Japan) and hemoglobin measurements were taken by photometric method. Total cholesterol was measured by the homogeneous enzymatic colorimetric method (Hitachi Modular P800 autoanalyzer, Roche Diagnostics Corp., Indianapolis, IN, USA) and low-density lipoprotein (LDL) cholesterol levels were determined with Friedewald’s method.^11^ Serum cardiac troponin I (cTn-I) levels were measured on a Dimension analyzer (Dade Behring Diagnostics, Amersfoort, the Netherlands) with a one-step enzyme immunoassay method based on the sandwich principle.

Measurements of MMP-2 were repeated twice. Previously frozen serum were allowed to thaw on ice and MMP-2 values were subsequently analyzed with the assistance of a bead-based multiplex immunoassay system (Bio-Plex Pro Human Inflammation Panel, Bio-Rad Laboratories, Hercules, CA, USA). To measure and quantify the development of selected sandwich immunocomplexes, the Bio-Plex MAGPIX System (Bio-Rad) was applied for the relevant bead sets. Final concentrations of analytes were determined with the help of Bio-Plex Manager v.5.0 software (Bio-Rad). Blood samples were taken at similar times in order to prevent the effect of daily rhythm on expression differences of inflammatory markers. Therefore, 46 patients who applied in the early hours (8:00-12:00 A.M.) were examined for MMP-2 expressions.

### Cardiac magnetic resonance imaging

In the process of obtaining the CMRI data, a single view with 4 chambers and cine short-axis sections (slice thicknesses of 6 mm at 10-mm intervals) was obtained, as well as a single view with 2 chambers. Evaluations of the LV systolic function indices were performed with the application of retrospective electrocardiogram gated turbo fast low angle shot (turbo-FLASH) sequences with echo time (TE) of 1.42 ms, repetition time (TR) of 39 ms, flip angle of 57°, and voxel size of 1.67 × 1.67 × 6 mm. The CMRI data obtained in this way were subsequently transferred in their entirety to a workstation. Thereafter, LV end-systolic volume (ESV) and LV end-diastolic volume (EDV) were determined by a reader employing Siemens syngo.via VA30 imaging software. In this process, the endocardial borders of the end-systolic and end-diastolic phases of short-axis stack images, which encompassed the LV within a range of space from the mitral annular line to the apex, were manually traced with exclusion of the papillary muscles. For cine images, the first phase was taken to be the end-diastolic phase, and the end-systolic phase was visually identified based on the cessation of inward LV motions.^12^ Infarct size was performed by the volume summation of hyperenhancement per-slice, and presented as percentage of total LV mass.

The definition of AR was applied in light of widely accepted threshold LVEDV values (∆LVEDV >12%).^13^

### T1 mapping

T1 maps were obtained both prior to and 15 minutes after the processing of gadolinium contrast. Three short-axial slices (apical, middle, and basal) were considered while obtaining the T1 mapping with the application of a modified look-locker inversion-recovery (MOLLI) investigational prototype sequence (Siemens Healthcare, Malvern, PA, USA)^14,15^ together with incorporation of an automatic registration algorithm as detailed in a previous publication.^16^ In the process of acquiring T1 MOLLI cardiac-gated data, a total of 3 inversion-recovery prepared look-locker experiments were carried out according to a single protocol.^14^ For the steps described here, the applied CMRI parameters included bandwidth of ~1090 Hz/pixel, flip angle of 35°, echo time of (TE) 1.1 ms, initial experimental T1 of 100 ms, TI increment of 80 ms, matrix of 192 × 124 pixels, spatial resolution of 2.2 × 1.8 × 8.0 mm, slice thickness of 8 mm, and scan time of 17 heartbeats.

### ECV measurement

Regions of interest (ROIs) were taken from the remote myocardium at a location 180° from the infarct zone, from the infarct zone across the full area of the recorded injury, and from the LV blood pool. These specified ROIs were copied with the application of T1 mappings before and after administering a contrast agent. In this process, manual corrections were also applied to maintain the margins of separation from tissue interfaces. ECV values were calculated based on the ratios of relevant T1 infarct area values previously obtained before and after administering the contrast agent in each ROI. As a result, registrations among T1 maps were not necessary to obtain ECV calculations with accuracy. Equation (1) was used here for ECV, wherein λ = ΔR1_myocardium_/ΔR1_blood_, ΔR1 = R1_post-contrast_ – R1_pre-contrast_, and R1 = 1/T1. Hematocrit (HCT) was also evaluated while these scans were being performed.


(1) ECV=(1−HCT)×λ

Matrix volume index were calculated by the product of LV myocardial volume (LV mass divided by specific gravity of myocardium [1.05 g/mL]) and ECV or (1 – ECV) for cellular volumes index.^17^ Examples of these measurements of T1 maps, and ECV and compartments are provided in Figure 2.

**Figure 2:** T1 maps and ECV values post-MI periods. CVi: cell volume index; ECV: extracellular volume; MVi: matrix volume index.

### Statistical analysis

Categorical variables were presented as numbers and percentages, and comparisons between groups were performed using chi-square, Yates correction, and Fisher tests. The normal distribution of numerical variables was evaluated with Kolmogorov-Smirnov tests and results with normal distribution were shown as mean±standard deviation while those with non-normal distribution were shown as median (interquartile range (IQR)). Intergroup comparisons of numerical variables were performed with Student t-tests or Mann-Whitney U tests. The change in CMRI parameters between 2 weeks and 6 months was evaluated with the paired T test or Wilcoxon test according to the normality distribution. To compare the considered CMRI parameters, a mixed model for repeated measures (MMRM) was established with the aim of comparing CMRI parameters and levels of MMP-2 between the groups in the post-MI period. The correlation between numerical variables were tested by Spearman correlation analysis. Effects on AR were evaluated by conducting univariable logistic regression analysis. Potential risk factors associated with AR (p<0.25) were included in these multivariable logistic regression models.^18,19^ In stepwise multivariable regression models, potential risk factors and changes of CMRI parameters from 2 weeks to 6 months post-MI were included. Receiver operating characteristic (ROC) curve analysis was conducted to establish a diagnostic discrimination of T1 mapping parameters on AR. IBM SPSS Statistics (IBM Corp., Armonk, NY, USA) was used for all analyses and p<0.05 (*) was accepted as statistically significant.

## Results

A total of 567 patients who were admitted to the emergency department with the diagnosis of STEMI were evaluated (mean age: 56.7±15.2 years, 14.3% women). Ninety two patients who met the exclusion criteria and evaluated for CMRI were included in the analysis (mean age: 54.1±9.0 years), and patients were mostly male (90.2%) with a representative risk profile for cardiovascular disease. Demographic, clinical and CMRI characteristics are shown in Tables 1 and 2. AR was detected in 32.6% (n=30) of all patients at 6-months after MI. The median cardiac troponin I, and median high-sensitivity C-reactive protein levels were higher in the AR group compared to without AR group. There was no significant difference between the demographic and other clinical characteristics of the patients with and without AR groups (Table 1).

**Table 1:** Demographic and laboratory findings

Variables			Total population n=92	Adverse remodeling		p
				Yes n=30	No n=62	
Demographic findings						
	Age, years		54.1±9.0	53.4±8.5	54.4±9.3	0.623
	Male gender, n(%)		83(90.2)	25(83.3)	58(93.5)	0.241
	BMI, kg/m^2^		26.7±4.3	27.0±3.7	26.5±4.6	0.704
	BSA, m^2^		1.9±0.2	1.9±0.2	1.9±0.2	0.997
	Hypertension, n (%)		40(43.5)	12(40.0)	28(45.2)	0.661
	Diabetes, n (%)		27(29.3)	9(30.0)	18(29.0)	0.999
	Dyslipidemia, n (%)		24(26.1)	10(33.3)	14(22.6)	0.315
	Smoking, n (%)		46(50.0)	19(63.3)	27(43.5)	0.113
**Clinical findings**						
	Heart rate, bpm		76.9±16.8	75.2±12.6	77.8±18.7	0.509
	SBP, mm Hg		123±15.5	124.1±14.7	122.4±16.1	0.657
	DBP, mm Hg		77.2±12.2	77.8±11.2	77.0±12.8	0.785
	Symptom-to-balloon time, mins		312.2±68.4	304.6±67.2	317.4±69.8	0.535
	Door-to-balloon time, mins		28.1±8.8	27.2±8.4	29.5±9.0	0.358
	IRA, n(%)					
		LAD	67(72.8)	22(73.3)	45(72.6)	0.999
		Cx	25(27.2)	8(26.7)	17(27.4)	
	Grace score		128.5±30.4	131.1±24.0	127.3±33.2	0.529
	Pre-PCI TIMI flow, n(%)					
		0	54(58.7)	18(60.0)	36(58.1)	0.848
		1	15(16.3)	5(16.7)	10(16.1)	
		2	2(17.5)	5(16.7)	12(19.4)	
		3	6(6.5)	2(6.7)	4(6.5)	
	Post-PCI TIMI flow >2, n(%)		90(97.8)	29(96.7)	61(98.4)	0.999
**Laboratory findings**						
	cTn-I, ng/L		46.4(37.7-57.8)	56.5(50.4-60.0)	41.7(35.5-48.0)	<0.001
	Hemoglobin, g/dL		13.8±1.5	14.0±1.8	13.6±1.4	0.375
	WBC, x10^9^/L		12.3±3.3	12.4±3.2	12.2±3.4	0.829
	Lymphocytes, x10^9^/L		2.3±0.8	2.2±0.8	2.4±0.8	0.149
	Neutrophils, x10^9^/L		8.4±2.1	8.8±1.9	8.2±2.2	0.190
	Monocyte, x10^9^/L		0.7±0.2	0.8±0.2	0.7±0.2	0.884
	Platelets, x10^9^/L		301.7±60.7	318.6±54.0	293.5±62.5	0.062
	Glucose, mg/dL		112(75-140)	114(100-149)	107(83-139)	0.390
	Total cholesterol, mg/dL		197(160-220)	191(155-211)	200(164-240)	0.264
	LDL, mg/dL		132(100-157)	119(101-144)	136(100-170)	0.261
	HDL, mg/dL		41.3±9.2	41.7±9.5	41.1±9.1	0.781
	C-reactive protein, mg/L		24.2(13-31.3)	28.0(16-41.2)	18.3(11.7-26.3)	0.024
**Discharge therapy, n(%)**						
	ACE/ARB		90(97.8)	30(100.0)	60(96.8)	0.816
	Beta blockers		90(97.8)	29(96.7)	61(98.4)	0.999
	Statins		91(98.9)	30(100.0)	61(98.4)	0.999

Numerical variables are shown as mean±standard deviation or median (IQR). Categorical variables are shown as number (%). ACE: angiotensin-converting enzyme; ARB: angiotensin II receptor blocker; BMI: body mass index; BSA: body surface area; Cx: circumflex artery; DBP: diastolic blood pressure; HDL: high-density lipoprotein; IRA: infarct-related artery; LAD: left anterior descending artery; LDL: low-density lipoprotein; PCI: percutaneous coronary intervention; SBP: systolic blood pressure; TIMI: thrombolysis in myocardial infarction.

**Table 2:** Acute and follow-up CMR results

Variables			Total population n=92	Adverse remodeling		p
				Yes n=30	No n=62	
**Second weeks**						
	LVEF, %		46.8±9.6	46.5±9.6	47.0±9.6	0.818
	LVEDV, mL		155(130.1-172.5)	153(135-176.7)	157.6(129-170)	0.723
	LVESV, mL		83.4(60.1-112.5)	93.5(70.7-128)	78.3(60-102)	0.207
	LVMi, g/m^2^		144(130-165)	147(133-176)	143(128-162)	0.257
	Infarct size, % of LV		15(11-22)	18(12-21)	15(10-21)	0.407
	Native T1, ms					
		Pre-contrast	1411.0±148.8	1421.2±162.8	1406.5±142.8	0.692
		Post-contrast	490.8±88.2	493.5±90.7	489.5±87.7	0.837
	ECV, %		40.1±7.4	42.9±6.4	39.3±8.2	0.037
	MVi, mL/m^2^		59.5±14.9	65.2±13.7	56.7±14.7	0.010
	CVi, mL/m^2^		88.0±15.0	86.3±13.6	88.9±15.6	0.447
**Sixth months**						
	LVEF, %		47.7±9.7	42.9±10.3	50.0±8.5	0.001
	LVEDV, mL		155.4(130-180.9)	180.7(159-227)	140(125.6-162.3)	<0.001
	LVESV, mL		79(59.7-116.1)	115.6(80-164)	68.9(54.4-90.7)	<0.001
	LVMi, g/m^2^		126(116-144)	138(122-166)	123(112-137)	0.002
	Infarct size, % of LV		12(8-16)	15(10-18)	11(7-15)	0.035
	Native T1, ms					
		Pre-contrast	1309.4±135.7	1325.2±117.0	1302.4±136.7	0.490
		Post-contrast	455.3±82.1	438.7±69.4	463.3±86.9	0.179
	ECV, %		45.8±6.2	49.7±6.1	44.0±5.4	<0.001
	MVi, mL/m^2^		60.5±15.2	70.7±12.1	55.6±11.8	<0.001
	CVi, mL/m^2^		70.6±11.0	70.9±12.2	70.4±10.4	0.851

Numerical variables are shown as mean±standard deviation and median (IQR). CVi: cell volume index; ECV: extracellular volume; LVEDV: left ventricular end-diastolic volume; LVEF: left ventricular ejection fraction; LVESV: left ventricular end-systolic volume; LVMi: left ventricular mass index; MVi: matrix volume index.

In the acute post-MI period, mean T1 levels in the infarct zone myocardium was not significantly different between the with and without AR groups. Mean ECV and mean MVi levels were higher in AR group compared to without AR group. At 6 months post-MI, mean ECV and mean MVi levels were higher in AR group compared to without AR group (Table 2).

Dynamic changes in CMRI at 6 months post-MI were summarized in Table 3. Accordingly, there was a similar reduction in native T1 values in the infarct zone myocardium after 6 months in with and without AR groups, while there was higher increase of ECV levels in AR group. MVi levels was significantly increased after 6 months in patients AR group, while there was not significantly different in without AR group. CVi levels was significantly decreased in both AR and without AR groups, and this decrease was similar between groups.

**Table 3:** Dynamic changes in injured myocardium according to presence of adverse remodeling

Variables	Adverse remodeling	Second weeks	Sixth months	p^1^	p^2^
LVMi, g/m^2^	No	143(128-162)	123(112-137)	<0.001	0.011
	Yes	147(133-176)	138(122-166)	<0.001	
Infarct size, % of LV	No	15(10-21)	11(7-15)	<0.001	0.715
	Yes	18(12-21)	15(10-18)	<0.001	
Native T1 infarct, ms	No	1406.5±142.8	1302.4±136.7	<0.001	0.378
	Yes	1421.2±162.8	1320.2±117	0.005	
ECV, %	No	39.3±8.2	44.0±5.4	<0.001	0.027
	Yes	42.9±6.4	49.7±6.1	<0.001	
MVi, mL/m^2^	No	56.7±14.7	55.6±11.8	0.480	0.007
	Yes	65.2±13.7	70.7±12.1	<0.001	
CVi, mL/m^2^	No	88.9±15.6	70.4±10.4	<0.001	0.164
	Yes	86.3±13.6	70.9±12.2	<0.001	

Numerical variables are shown as mean±standard deviation or median (IQR)

1 p Second weeks vs sixth months within remodeling groups.

2 p Comparison of the changes in follow-up (Adverse remodeling groups: No vs. Yes). CVi: cell volume index; ECV: extracellular volume, LVMi: left ventricular mass index; MVi: matrix volume index.

In regression model I examining the relationship between AR and dynamic changes at 6 months post-MI, an increased ΔLVMi levels and increased ΔECV levels were independently predictor of AR. In regression Model II, MVi and CVi derived from them were added instead of LVMi and ECV. Accordingly, an increased ΔMVi levels was independently predictor of AR. Model II had higher performance in explaining AR possibility compared to Model I (Model I: Nagelkerke R^2^=0.537 vs Model II: Nagelkerke R^2^=0.615) (Table 4). In addition, ΔMVi had superior diagnostic performance compared to ΔECV and ΔLVMi in predicting AR (Figure 3).

**Table 4:** Multivariable associations of T1 mapping parameters with adverse remodeling at six months post-MI

Variables		Univariable			Multivariable		
		OR	95% CI	p	OR	95% CI	p
**Model I**							
	cTn-I	1.05	1.01-1.09	0.011	1.28	1.05-1.55	0.013
	C-reactive protein	1.07	1.01-1.12	0.017	1.15	1.01-1.32	0.044
	ΔLVMi	1.28	1.12-1.48	0.010	1.36	1.14-1.78	0.012
	ΔInfarct size	1.02	0.98-1.06	0.249	-	-	-
	ΔNative T1 infarct	1.01	0.97-1.06	0.154	-	-	-
	ΔECV	1.04	1.01-1.08	0.025	1.05	1.02-1.09	0.041
					Nagelkerke R^2^=0.537, p<0.001		
**Model II**							
	cTn-I	1.05	1.01-1.09	0.011	1.32	1.12-1.56	0.014
	C-reactive protein	1.07	1.01-1.12	0.017	1.15	1.03-1.30	0.020
	ΔInfarct size	1.02	0.98-1.06	0.249	-	-	-
	ΔNative T1 infarct	1.01	0.97-1.06	0.154	-	-	-
	ΔMVi	1.06	1.01-1.11	0.004	1.03	1.01-1.06	0.010
	ΔCVi	0.97	0.95-0.99	0.089	-	-	-
					Nagelkerke R^2^=0.615, p<0.001		

Co-founder factor, including age, male gender, smoking, lymphocytes, neutrophils and platelet parameters, were adjusted in all analysis. Δ: the change of T1 mapping parameters from 2 weeks to 6 months post-MI. CI: confidence interval; CVi: cell volume index; ECV: extracellular volume; LVMi: left ventricular mass index; MVi: matrix volume index; OR: odds ratio.

**Figure 3:** Diagnostic performance of ΔECV, ΔLVMi and ΔMVi in predicting AR. AUC: area under the curve; ΔAUC: difference of area under the curve; CI: confidence interval; ECV: extracellular volume; LVMi: left ventricular mass index; MVi: matrix volume index; SE: standard error.

Median MMP-2 levels at first day post-MI was higher in AR group compared to without AR group [33241.6 (IQR: 18811.3-60196.5) vs 21333 (IQR: 16043.3-28784.3) pq/mL, p=0.026], while there was not significantly different at 2 weeks post-MI [32811.3 (IQR: 19906.7-51487.2) vs 25572.8 (IQR: 16831-46611.6) pq/mL, p=0.340]. Median MMP-2 levels was not significantly different at 2 weeks compared to the first day post-MI in AR group (33241.6 vs 32811.3 pq/mL, p=0.809), while increased in without AR group (21333 vs 25572 pq/mL, p=0.046). A positive correlation was found between baseline levels of MMP-2 and baseline level of LVMi, ECV and MVi (Figure 4) (Table 5).

**Table 5:** The relationship between matrix metalloproteinases-2 and CMR parameters

Variables		First day post-MI MMP-2		2 weeks post-MI MMP-2	
		r	p	r	p
**2 weeks**					
	LVMi	0.301	0.047	0.089	0.551
	Infarct size	0.024	0.878	0.221	0.144
	Native T1 infarct	0.135	0.367	0.166	0.263
	ECV (%)	0.535	<0.001	0.355	0.014
	MVi	0.549	<0.001	0.325	0.029
	CVi	0.031	0.837	0.143	0.338

CVi: cell volume index; ECV: extracellular volume; LVMi: left ventricular mass index; MMP-2: matrix metalloproteinases-2; MVi: matrix volume index.

**Figure 4:** Relationship between matrix metalloproteinase-2 and ECV (A), VMi (B), and CVi (C). AR: adverse cardiac remodeling; CVi: cell volume index; ECV: extracellular volume; MVi: matrix volume index.

## Discussion

The main findings of this study were that, in acute STEMI patients who developed AR at the 6-month follow-up, 1) The ECV values increased more prominently after 6 months, 2) This increase was in the direction of the matrix volume, 3) A positive correlation was found between MMP-2 levels and ECV and MVi levels, 4) ΔMVi in 6-month follow-up was superior to ΔECV in predicting AR, 5) The regression model in which MVi was included was superior in explaining AR.

Quantitative T1 mapping measures pixel-based T1 relaxation time in the myocardium. T1 relaxation time varies depending on the differentiation around the tissue and it reflects pathological processes at tissue level.^20^ In patients with heart failure, T1 relaxation times have been reported to be positively correlated with fibrosis detected by biopsy.^17^ Increased amounts of myocardial fibrosis disrupt the structure of the myocardium and cause systolic and diastolic dysfunction.^21^ This is characterized by the excessive accumulation of ECM proteins. Increases in total myocardial water, edema, and collagen deposition due to inflammatory response result in increased native T1 values.^22^ Decreases in native T1 values in STEMI patients, regardless of AR development, may be associated with preserved healing capacity, resorption of edema, and necrotic tissue in the infarcted myocardium. Segmental fibrosis develops in the area of necrosis, and replacement or interstitial fibrosis develops in non-necrotic areas after MI. High ECV values reflect excessive collagen deposition, scarring, and extensive interstitial fibrosis^5^ and may be important indicators of AR.^23^ This is consistent with the fact that ECV was an independent predictor in the regression model I established in the present research. Moreover, the elimination of native T1 supports the idea that the extracellular space has a more prognostic role in AR development.^22,24^

T1 mapping has introduced a new concept in cardiology practice by allowing the myocardium to be separated into its cellular compartment (mostly myocytes) and interstitial compartment (mostly collagen or edema). The ECV reflects a relative volume ratio of total myocardial volume. However, it does not sensitively reflect dynamic changes in myocardial tissue when change of cellular and extracellular components.^17^ Thus, evaluating ECV by dividing it into matrix and cell volume indices can provide more detailed information about the mechanism of AR and make the new paradigms of cardiac vulnerability easier to understand and apply. In previous studies of different cardiac diseases, enlarged cell and matrix models were established, a decrease in their levels was detected with medical treatment as per the relevant guidelines, and the decrease was associated with improvement in cardiac volume and functions.^7,17^ In our study, although the symptom to balloon time did not differ in patients who developed and did not develop AR post MI, and all patients received guideline treatment, and a significant decrease was found in cell volume index. In addition, there was no significant difference in infarct size in the second week between the two study groups. However, similar reduction in CVi was observed among those who developed AR despite receiving similar treatment, but high incremental variation in the MVi was concluded to be an independent predictor of AR. This finding is consistent with the mechanisms through which myocyte recovery may precede adaptive remodeling in the extracellular component.^7,25^ In addition, this may show that cell volumes normalize earlier than matrix volumes. On the other hand, high MMPs levels may contribute to the late normalization of matrix volume, which may be associated with a later improvement in infarct size at 6 months in patients with AR.^26^

The regression model including ECV showed less diagnostic performance compared to the regression model including ECV components. On the other hand, the lower diagnostic performance of ECV compared to both cellular and extracellular components in ROC analysis supports that ECV may be less sensitive to tissue changes. The superior diagnostic performance of MVi in predicting AR compared to ECV and CVi may indicate that it may be more sensitive to tissue changes. All of the findings presented here highlight the importance of cardiac fibroblasts in changes in the dynamic structure of the extracellular matrix, including diffuse fibrosis (matrix volume).^27^ The association of cardiac fibroblasts with changes in the collagen cycle highlights the dynamic nature of the extracellular matrix. MVi quantification could add more predictive information and support reversibility in AR development, especially considering its association with MMPs. In clinical practice, MVi can provide detailed information on the mechanism of AR by better reflecting myocardial tissue changes. Therefore, MVi may be a guide in terms of unfavorable prognosis after STEMI. It may also be important in terms of endpoints.^17^ Because both focal fibrosis and extensive fibrosis have been shown to be univariate predictors of outcome.^28^ Previous research has shown that cardiac fibroblasts constitute 60-70% of all myocardial cells, exerting crucial influence in processes of myocardial repair to ensure the continuation of post-injury cardiac functions. Enhanced pro-fibrotic and pro-inflammatory profiles might lead to gradual increases in the stiffness of the myocardium as well as decreased myocardial compliance alongside ventricular systolic and diastolic dysfunction. This process has its foundation in the early activation of MMPs.^29,30^ MMP-2 can be self-activated in the ECM by the action of free radicals produced by activated tissue macrophages.^31^

An excessive inflammatory response may result in excessive production of MMP-2, and this, in turn, may play a role in AR by causing cardiac vulnerability.^32^ The mechanism of this process may be oriented toward ECV enlargement (mainly with increases in matrix volume) as a result of excessive MMP-2 production due to excessive inflammatory response. Therefore, MMP activation may represent a viable therapeutic target for regulating ECM transformation during the pathological process of AR development after MI.

This study, which represents a cohort of patients who had experienced STEMI for the first time, has certain limitations. Although the findings of this small sample are in line with the literature, a larger sample could provide more consistent results. On the other hand, the pathophysiology of AR is complex. Changes in the remote zone myocardium, microRNAs, and cytokines may play important roles, and these could not be evaluated in this study. The consideration of these factors in future studies could further illuminate the association of change in the compartments of ECV with inflammation.

## Conclusion

This study provide further evidence for the pathophysiological significance of tissue characteristics and LV remodeling in the setting of acute STEMI. We conclude that change of matrix volume, superior to change of ECV, after acute MI period were independently predictor of AR, reflecting increased interstitial fibrosis. Increased levels of MMP-2 at the onset of acute MI play an important role in the change in matrix volume, and therefore may be a therapeutic target.
